# DNAH5‐Associated Primary Ciliary Dyskinesia With Natural Paternity and Advanced Bronchiectasis

**DOI:** 10.1002/rcr2.70836

**Published:** 2026-11-10

**Authors:** Sonja Momchilovikj, Angela Debreshlioska, Rabije Mustafa, Irina Angelovska, Kristina Dimitrijevikj, Berna Omerova

**Affiliations:** ^1^ University Clinic of Pulmonology and Allergology Skopje North Macedonia; ^2^ Clinical Hospital Bitola Bitola North Macedonia

**Keywords:** bronchiectasis, *DNAH5*, Kartagener syndrome, primary ciliary dyskinesia, situs inversus totalis

## Abstract

Primary ciliary dyskinesia (PCD) is a rare inherited disorder of motile cilia that may remain unrecognised until adulthood. We report a 43‐year‐old lifelong non‐smoker with recurrent respiratory infections since childhood, chronic rhinosinusitis, situs inversus totalis, advanced bilateral bronchiectasis, severe non‐reversible airflow obstruction, and chronic hypoxemic respiratory failure. His course was complicated by chronic *Pseudomonas aeruginosa* airway infection and frequent exacerbations. Despite a characteristic Kartagener phenotype, he had fathered six biological children conceived without assisted reproductive techniques. Whole‐exome sequencing identified a homozygous pathogenic frameshift variant in *DNAH5* (NM_001369.3:c.5563dup; p.(Ile1855Asnfs*6)), establishing the molecular diagnosis. This case highlights the reproductive phenotypic variability of *DNAH5*‐associated PCD and emphasises that natural paternity does not exclude PCD or justify delaying molecular genetic evaluation.

## Graphical abstract

Primary ciliary dyskinesia (PCD) is a rare, genetically heterogeneous disorder of motile cilia that may cause chronic sinopulmonary disease, bronchiectasis, and laterality abnormalities. The combination of bronchiectasis, chronic rhinosinusitis, and situs inversus is traditionally referred to as Kartagener syndrome. We report an adult with advanced DNAH5‐associated PCD and natural paternity of six biological children.

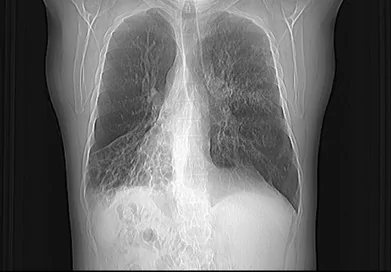

## 1 Introduction

Primary ciliary dyskinesia (PCD) is a rare, genetically heterogeneous disorder of motile cilia that may cause chronic sinopulmonary disease, bronchiectasis, and laterality abnormalities. The combination of bronchiectasis, chronic rhinosinusitis, and situs inversus is traditionally referred to as Kartagener syndrome [1]. Biallelic pathogenic variants in DNAH5, which encodes a heavy‐chain component of the outer dynein arm, are an established cause of autosomal recessive PCD [2]. Molecular testing can establish the underlying genetic aetiology, particularly in adults with delayed diagnostic confirmation or atypical phenotypic features [1]. Male infertility is common in PCD because abnormalities affecting motile respiratory cilia may also impair sperm flagellar function; however, reproductive involvement is variable [3, 4]. We report an adult with advanced DNAH5‐associated PCD and natural paternity of six biological children.

## 2 Case Report

A 43‐year‐old lifelong non‐smoker with no relevant occupational exposure was evaluated for chronic productive cough, progressive dyspnoea, and recurrent respiratory tract infections. He had experienced frequent upper and lower respiratory tract infections since childhood. Since 2017, symptoms had progressively worsened, with daily yellow‐green sputum production, intermittent low‐grade fever, and occasional haemoptysis. Bilateral bronchiectasis and situs inversus totalis were identified in 2017, when a clinical diagnosis of Kartagener syndrome was considered. Confirmatory PCD testing, including genetic testing, was not performed at that time because of technical and financial constraints, and the diagnosis remained presumptive until molecular testing became available. A formal PICADAR score was not recorded, as this screening tool was not routinely incorporated into the diagnostic pathway at our centre at that time. In 2021, microbiologically confirmed pulmonary tuberculosis was diagnosed and successfully treated. During the preceding 12 months, he had experienced five respiratory exacerbations, three requiring hospitalisation. He had fathered six biological children conceived without assisted reproductive techniques. There was no family history of bronchiectasis, laterality abnormalities, or infertility.

His medical history also included bilateral nasal polyposis, chronic pansinusitis, sinus tachycardia, and persistent non‐reversible airflow obstruction. He had been repeatedly evaluated by ENT specialists since 2017. In 2022, functional endoscopic sinus surgery, septoplasty, and polypectomy were recommended during separate assessments, but the patient declined the proposed procedures despite counselling. Conservative management included intranasal corticosteroid therapy. With subsequent deterioration in respiratory status, elective ENT surgery was considered high risk.

He was receiving long‐term oxygen therapy at 1.5–2 L/min. During an acute exacerbation, oxygen saturation was approximately 60% on room air and improved to 86%–90% with supplemental oxygen. Laboratory testing showed mild leukocytosis (12 × 10^9^/L), neutrophilia (84%), and a C‐reactive protein level of 15 mg/L. Arterialised capillary blood gas analysis demonstrated hypoxemic respiratory failure without hypercapnia (pO_2_ 6.17 kPa; pCO_2_ 5.67 kPa). Post‐bronchodilator spirometry showed severe non‐reversible airflow obstruction, with FEV_1_ 37% predicted, FVC 51% predicted, and an FEV_1_/FVC ratio of 0.64. Residual volume was 164% predicted, indicating marked air trapping, while diffusing capacity was preserved.

Chest imaging demonstrated dextrocardia, situs inversus totalis, diffuse bilateral mosaic attenuation, and extensive cystic and tubular bronchiectasis. Changes were most pronounced in the left upper lobe and lingula and posterior right lower lobe, with bronchial wall thickening, intraluminal secretions, and adjacent fibrotic and inflammatory parenchymal abnormalities (Figures 1 and 2).

**FIGURE 1:**
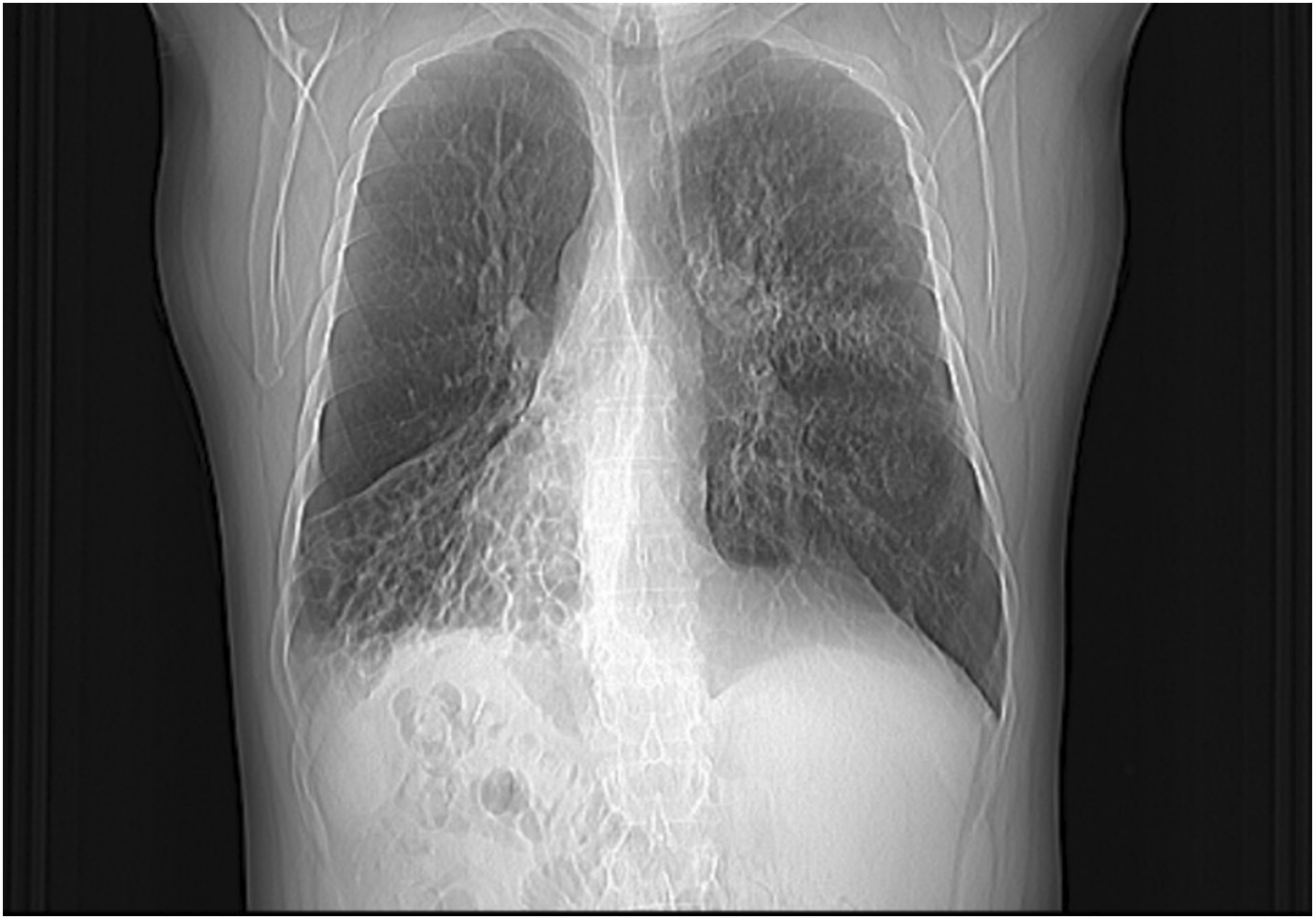
Frontal chest radiograph demonstrating dextrocardia and bilateral coarse bronchiectatic changes.

**FIGURE 2:**
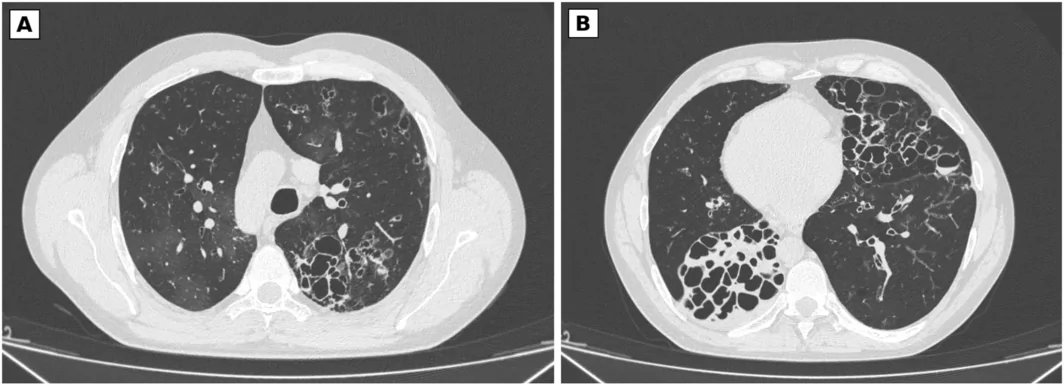
Chest computed tomography findings. (A) Axial chest CT showing extensive cystic and tubular bronchiectasis predominantly involving the left upper lobe. (B) Axial chest CT showing dextrocardia and extensive bilateral cystic bronchiectasis, most pronounced in the right lower lobe and lingula.

Bronchoalveolar lavage performed in 2017 yielded mucoid *Pseudomonas aeruginosa* . An initial eradication attempt with intravenous ceftazidime and amikacin was followed by long‐term inhaled colistin. *P. aeruginosa* was repeatedly recovered from subsequent respiratory specimens, consistent with chronic airway infection. Follow‐up sputum smear microscopy, mycobacterial culture, and Xpert MTB/RIF showed no evidence of recurrent or active mycobacterial infection. Sweat chloride was < 30 mmol/L, making cystic fibrosis unlikely, and *Aspergillus fumigatus* sensitisation was negative.

The combination of childhood‐onset sinopulmonary disease, chronic sinonasal disease, extensive bronchiectasis, and situs inversus totalis remained strongly suggestive of PCD with a Kartagener phenotype. In September 2025, whole‐exome sequencing identified a homozygous pathogenic frameshift variant in DNAH5 (NM_001369.3:c.5563dup; p.(Ile1855Asnfs*6)), exon 34, establishing the molecular diagnosis of DNAH5‐associated PCD.

Management included airway‐clearance therapy, inhaled hypertonic saline, long‐term oxygen therapy, inhaled colistin, and susceptibility‐guided systemic antibiotics during exacerbations. Long‐term azithromycin 500 mg three times weekly was introduced during the most recent year because of recurrent bronchiectasis exacerbations. Triple inhaled therapy (ICS/LABA/LAMA), previously initiated for severe persistent non‐reversible airflow obstruction under a working diagnosis of COPD, was continued; no concomitant asthma had been documented. During 9 months of follow‐up after molecular diagnosis, lung function remained stable, although exacerbation frequency did not decrease in the context of inconsistent adherence to inhaled colistin. Lobectomy was considered but not undertaken because bilateral disease, severe airflow obstruction, and chronic hypoxemic respiratory failure conferred a high perioperative risk.

## 3 Discussion

This case demonstrates delayed molecular confirmation of PCD despite a characteristic clinical phenotype. Kartagener syndrome had been clinically suspected in 2017 on the basis of bronchiectasis, chronic sinonasal disease, and situs inversus totalis; however, confirmatory testing was not performed at that time. By the time molecular confirmation was obtained, the patient had developed advanced bronchiectasis, severe non‐reversible airflow obstruction, chronic hypoxemic respiratory failure, frequent exacerbations, and chronic *Pseudomonas aeruginosa* airway infection. This highlights the importance of completing the diagnostic evaluation when PCD is clinically suspected, even in adulthood.

Whole‐exome sequencing identified a homozygous pathogenic frameshift variant in DNAH5. DNAH5 encodes an outer dynein arm heavy‐chain component, and biallelic pathogenic variants are an established cause of autosomal recessive PCD [2]. Molecular testing established the aetiology when nasal nitric oxide measurement and specialised ciliary functional or ultrastructural testing were unavailable [1]. The absence of these investigations represents a limitation; however, the characteristic clinical phenotype combined with the homozygous pathogenic DNAH5 variant provided molecular confirmation.

The patient's natural paternity of six biological children illustrates reproductive phenotypic variability in DNAH5‐associated disease and demonstrates that preserved reproductive capacity does not exclude PCD [3, 4]. Because semen analysis was not performed, normal sperm motility cannot be inferred from paternity alone.

Chronic *P. aeruginosa* airway infection despite an initial eradication attempt and subsequent inhaled colistin reflected advanced structural lung disease and likely contributed to the persistent exacerbation burden [5]. The previous pulmonary tuberculosis may also have contributed to structural lung damage but could not explain the childhood‐onset sinopulmonary disease, chronic sinonasal involvement, and situs inversus totalis. Overall, this case demonstrates that delayed confirmation of PCD may be associated with substantial respiratory morbidity and that an atypical reproductive phenotype should not delay molecular genetic evaluation.

## Author Contributions

**Sonja Momchilovikj:** conceptualization, investigation, writing – original draft, methodology, writing – review and editing, formal analysis, resources. **Angela Debreshlioska:** conceptualization, investigation, visualization, writing – review and editing, writing – original draft, supervision. **Rabije Mustafa:** visualization, investigation, project administration, supervision. **Irina Angelovska:** conceptualization, supervision, validation, visualization, funding acquisition, writing – original draft. **Kristina Dimitrijevikj:** conceptualization, formal analysis, visualization, writing – review and editing. **Berna Omerova:** data curation, project administration, software, writing – review and editing.

## Funding

The authors have nothing to report.

## Consent

The authors declare that written informed consent was obtained for the publication of this manuscript and accompanying images using the form provided by the Journal.

## Conflicts of Interest

The authors declare no conflicts of interest.

## Data Availability

The data that support the findings of this study are available from the corresponding author upon reasonable request.
